# Differences in neural responses to ipsilateral stimuli in wide-view fields between face- and house-selective areas

**DOI:** 10.1371/journal.pone.0192532

**Published:** 2018-02-16

**Authors:** Bin Wang, Ting Li, Yan Niu, Jie Xiang, Junjie Cheng, Bo Liu, Hui Zhang, Tianyi Yan, Susumu Kanazawa, Jinglong Wu

**Affiliations:** 1 College of Computer Science and Technology, Taiyuan University of Technology, Taiyuan, Shanxi, China; 2 Department of Radiology, First Hospital of Shanxi Medical University, Taiyuan, Shanxi, China; 3 School of Life Science, Beijing Institute of Technology, Beijing, China; 4 Graduate School of Medicine, Dentistry, Pharmaceutical Sciences, Okayama University, Okayama, Japan; 5 Graduate School of Natural Science and Technology, Okayama University, Okayama, Japan; 6 Key Laboratory of Biomimetic Robots and Systems, Ministry of Education, Beijing Institute of Technology, Beijing, China; Tokai University, JAPAN

## Abstract

Category-selective brain areas exhibit varying levels of neural activity to ipsilaterally presented stimuli. However, in face- and house-selective areas, the neural responses evoked by ipsilateral stimuli in the peripheral visual field remain unclear. In this study, we displayed face and house images using a wide-view visual presentation system while performing functional magnetic resonance imaging (fMRI). The face-selective areas (fusiform face area (FFA) and occipital face area (OFA)) exhibited intense neural responses to ipsilaterally presented images, whereas the house-selective areas (parahippocampal place area (PPA) and transverse occipital sulcus (TOS)) exhibited substantially smaller and even negative neural responses to the ipsilaterally presented images. We also found that the category preferences of the contralateral and ipsilateral neural responses were similar. Interestingly, the face- and house-selective areas exhibited neural responses to ipsilateral images that were smaller than the responses to the contralateral images. Multi-voxel pattern analysis (MVPA) was implemented to evaluate the difference between the contralateral and ipsilateral responses. The classification accuracies were much greater than those expected by chance. The classification accuracies in the FFA were smaller than those in the PPA and TOS. The closer eccentricities elicited greater classification accuracies in the PPA and TOS. We propose that these ipsilateral neural responses might be interpreted by interhemispheric communication through intrahemispheric connectivity of white matter connection and interhemispheric connectivity via the corpus callosum and occipital white matter connection. Furthermore, the PPA and TOS likely have weaker interhemispheric communication than the FFA and OFA, particularly in the peripheral visual field.

## Introduction

In the primate visual system, visual input to each cerebral cortical hemisphere comes largely from the contralateral visual field [1–3]. Neural responses to ipsilateral stimuli are negative or zero in V1–V3, but these responses begin to increase in higher-order visual areas. The extent of neural activity elicited by ipsilateral stimuli increases between V3A and hV4; cortical areas that are located superiorly toward the parietal cortex and anteriorly along the lateral occipital cortex exhibit greater ipsilateral responses [4, 5].

Several category-selective areas are found in the lateral and ventral visual cortex, such as the face-selective areas (fusiform face area, FFA, and occipital face area, OFA) [6–8] and the house-selective areas (parahippocampal place area, PPA, and transverse occipital sulcus, TOS) [9, 10]. More recently, several studies have demonstrated that these category-selective areas respond not only to preferred objects but also to non-preferred objects [11, 12]. Responses to the preferred object are greater than those to non-preferred objects.

These category-selective areas also exhibit various degrees of neural responses to both preferred and non-preferred stimuli in the ipsilateral visual field [13–16]. For example, the face-selective areas (FFA and OFA) exhibit strong activation to ipsilaterally presented images of faces and objects and much weaker activation to ipsilaterally presented images of scenes [14]. Recently, Choi et al. detected ipsilateral-dominant voxels in the right FFA [17]. The house-selective areas (PPA and TOS) exhibit strong activation to ipsilaterally presented images of scenes and much weaker activation to images of objects [15].

Moreover, these category-selective areas exhibit central-peripheral organization [18, 19]. The FFA represents foveal eccentricities, and the PPA represents peripheral eccentricities. In our recent study on neural responses to faces and houses presented in the peripheral visual field, we found that the processing strategies for the encoding of wide-view field visual information differs between the FFA and PPA [20]. Both face- and house-selective areas exhibit various degrees of neural responses to stimuli in the ipsilateral visual field [13–16]. We proposed that the distance between stimuli and the vertical meridian in a wide-view field can be much larger than the population receptive fields of category-selective areas [21–24], and the presentation of stimuli in a wide-view field provides an advantage over studies of the neural responses to stimuli in the ipsilateral visual field. However, due to the limitations of visual field size in MRI scans, the neural responses in the face- and house-selective areas that are evoked by ipsilateral stimuli in the peripheral visual field remain largely unclear.

**Fig 1:**
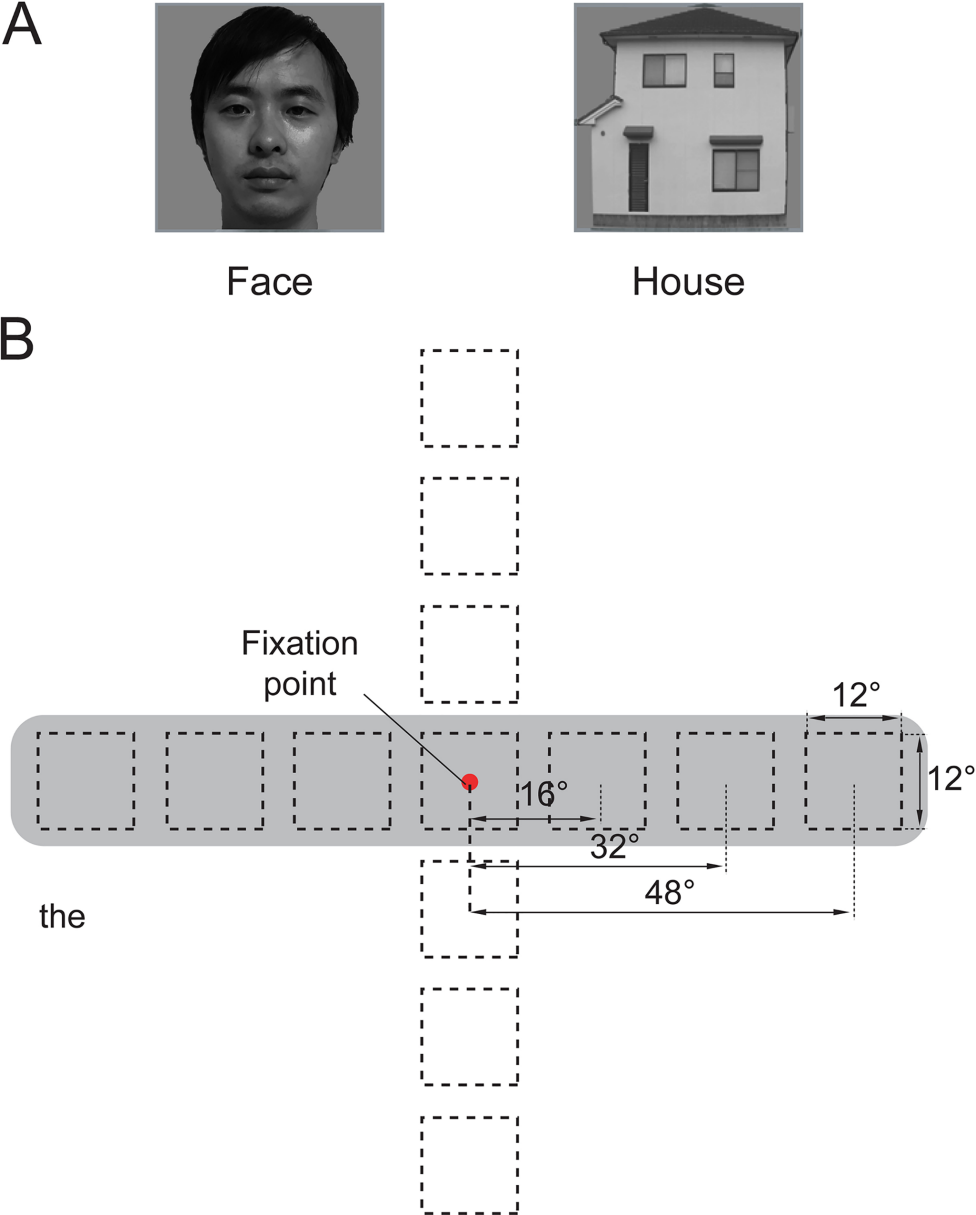
The stimulus configuration. (A) Example images of a face and a house are shown. The images of the faces shown here do not depict the actual stimuli and are intended only as examples. The individual in this manuscript has given written informed consent (as outlined in PLOS consent form) to publish these case details. (B) The size and position of the stimuli used in the position experiment. The images were either centered at the fixation point (0°) or centered at 16°, 32° or 48° for the left horizontal, right horizontal, upper vertical, or lower vertical meridians, respectively. The gray background indicates the position conditions used in the present study to investigate the contralateral-ipsilateral responses in the face- and house-selective areas.

In the present report, we investigated the ipsilateral neural responses elicited by stimuli presented in a wide-view field by analyzing MRI data from our previous fMRI studies [20, 25]. Subjects were instructed to categorize images of faces and houses that were displayed in 13 positions using a wide-view presentation field with 60° of eccentricity while maintaining fixation. We used the horizontal meridian conditions to investigate the contralateral and ipsilateral neural responses in face- and house-selective areas (Fig 1).

## Materials and methods

The data used in this paper are from a previous fMRI experimental study [20]. In the present paper, we used only the horizontal conditions to investigate the contralateral and ipsilateral neural responses. The details about this behavioral paradigm and data processing have been reproduced here.

### Subjects

All MR imaging was performed at the Hospital of Okayama University. The experiments were performed with the written consent of each subject and were approved by the Ethics Committee of the Hospital of Okayama University. Data from seven right-handed subjects (5 males, 2 females, aged 22–25 years) were used in the following analyses.

### Presentation of the stimuli

The stimuli were projected on a wide-view visual presentation system that had been upgraded from a previous version [25, 26]. The subjects viewed the stimuli on a hemisphere that was 52 mm in diameter, and the curvature radius of this hemisphere was 30 mm. Monocular (right eye) presentations were performed using the hemispheric screen. The subject’s eye was fixed on the central axis 3 mm away from the screen. The subjects wore contact lenses to focus on the stimulus, and the visual field of the stimulus was 120° horizontal × 120° vertical or 60° of eccentricity.

### Position experiments

The position experiments utilized grayscale images of human faces and houses. The face images were taken from the FEI face database (http://fei.edu.br/~cet/facedatabase.html), and the houses images were photos taken in Okayama City. The images of the faces shown in Fig 1 A do not depict the actual stimuli and are intended only as examples. The individual in this manuscript has given written informed consent (as outlined in PLOS consent form) to publish these case details. The objects were presented at a variety of positions and grayscale backgrounds (Fig 1A). The position experiments utilized 48 unique images from each category. The images subtended a 12° visual angle at each position. We chose to use a constant image size because the magnification factors in the face- and house-selected areas were unknown, and the magnifications at the center and periphery were quite different. We wished to compare the neural activation corresponding to the images of the faces and houses at different positions throughout the central and peripheral visual fields. The images were centered at the fixation point (0° eccentricity) and were centered at 16°, 32° and 48° of eccentricity along 4 meridians: the left horizontal meridian, right horizontal meridian, upper vertical meridian and lower vertical meridian. A total of 13 positions were arranged in the 4 levels of eccentricities (0°, 16°, 32° and 48°) for each meridian (Fig 1B).

The position experiments included 6 runs of a block design experiment. Each run contained one 8-s block for each position and category combination; thus, the session contained 26 image blocks per run (2 categories × 13 positions). The image blocks were interleaved with 8-s baseline blocks (a grayscale screen with a central fixation point). In each image block, 8 images obtained from 48 images per category (face or house) were presented at a specific position in a random order. The images were presented at a rate of 1 Hz (800 ms per image with a 200-ms inter-stimulus interval).

During the scanning process, the subjects were instructed to categorize each image while fixating on a red point. When the red disk dimmed, the subjects reported their categorization with two buttons that corresponded to either a face or a house. The dimming prompts lasted 1.2 s with a 1.8- or 3.8-s interval between the prompts. There were 2 or 3 prompts in one block. The fixation task was primarily used to ensure that the subjects maintained their fixation during the scans and paid attention to the entire visual field. Before scanning, the subjects were well practiced in this task to minimize false alarms and to maintain their focus on the fixation point. Behavioral responses were collected during the scanning with a magnet-compatible button box connected to the stimulus computer.

### Retinotopic mapping experiments

To identify the retinotopic areas of the visual cortex, a clockwise rotating wedge and expanding ring stimuli were employed [2, 3, 26]. These stimulus apertures contained 100% contrast black-and-white checkerboard patterns, and they phase-reversed at a temporal frequency of 8 Hz at an eccentricity that ranged from 2.4° to 60°. The wedge stimulus with an angle of 22.5° and an eccentricity of 2.4° to 60° was slowly rotated clockwise around a red fixation disk (approximately 1°) that was presented at the center of the stimulus. The wedge rotated at 22.5° steps and remained at each position for 6 s before moving to the next position. The eccentricity of the expanding rings ranged from 2.4° to 60°, and the width of the ring stimuli was expanded in exponential increments. The corresponding ring sizes were 1.2°, 1.8°, 2.7°, 4.0°, 6.0°, 9.0°, 13.4° and 20.0°. These expanding ring stimuli were moved in 8 discrete steps and remained at each position for 6 s before automatically expanding to the next position. All experiments involved passive viewing, and the subjects were required to maintain their gaze on the red fixation disk in the center of the screen that flickered at a temporal frequency of 4 Hz throughout the scan. Six complete cycles of rotations and checkerboard expansions were conducted.

### Image acquisition

Imaging was performed using a 3-tesla MR scanner (Siemens Allegra, Erlangen, Germany). For the functional series, we continuously acquired images with 30 slices using a standard T2-weighted echo-planar imaging (EPI) sequence (TR = 2 s; TE = 35 ms; flip angle = 85°; 6 4 × 6 4 matrices; in-plane resolution: 2.3 × 2.3 mm; slice thickness: 2 mm, with a gap of 0.3 mm). The slices were manually aligned approximately perpendicular to the calcarine sulcus to cover most of the occipital, posterior parietal and posterior temporal cortices. After the functional scans, high-resolution sagittal T1-weighted images were acquired using a magnetization-prepared rapid gradient echo sequence (MP-RAGE; TR = 1800 ms; TE = 2.3 ms; matrix 256 × 256 × 224; 1 mm isotropic voxel size) to obtain a 3D structural scan.

### Data preprocessing

Anatomical and functional images were analyzed using BrainVoyager QX 2.11 (Brain Innovation, Maastricht, The Netherlands). The anatomical scans were segmented for the identification of the white/gray matter boundaries and were then used for cortical surface reconstruction and inflation. In each functional run, the first 2 volumes were discarded to assure that a steady state was reached. The functional data were preprocessed with motion and scan-time correction and high-pass temporal filtering (0.01 Hz) before statistical analysis. Spatial smoothing using a full-width, half-maximum Gaussian kernel of 4 mm was applied to the position experiment’s data but not to the retinotopic mapping data. The functional data were transformed into the conventional Talairach space and resliced into 2-mm isotropic voxels.

### General linear model

The blood-oxygen-level-dependent (BOLD) signal of the fMRI data was analyzed voxel-by-voxel using a statistical test based on the general linear model (GLM). Our analysis consisted of a multiple regression analysis with a regressor for each condition in the experiment that used a boxcar shape and assumed a double-gamma hemodynamic response function. There was a condition for each category and each position, and in total 26 (2x13) conditions. After the coefficients for all regressors were computed, we performed t-tests between the coefficients for different conditions. For each subject, a fixed-effects analysis of variance was performed to combine the 6 runs of the position experiment. All statistical analyses used the statistical threshold of p < 0.05 with a false-discovery-rate (FDR) correction and a cluster threshold of 10 voxels. The analyses were performed on 3D voxels, and the activation maps were rendered on a flattened cortical surface for visualization.

### Retinotopic mapping analysis

Our retinotopic mapping experiments employed a standard phase-encoded retinotopy design [2, 3, 26]. For the polar angle and eccentricity mapping, the stimulation blocks were modeled with boxcar functions that were convolved with a double-gamma hemodynamic response function [27]. The stimulus-driven modulation of the BOLD response in each functional voxel was revealed via a linear correlation map analysis. This phase was mapped into physical units by identifying the stimulus parameter (polar angle or eccentricity) that corresponded to the time. The color-coded cortical areas were classified based on an r-value threshold of 0.25. To aid the visualization, the retinotopic maps were projected onto computationally flattened representations of the cortical surface.

### Region-of-interest analysis

For the accuracies at most peripheral position (eccentricity 48°) were less than the chance level (50%), and the rather weak neural activities in response to the objects at most peripheral positions, especially for the responses to ipsilateral objects, we main focused on the positons of eccentricity of 16° and 32° in ROI analysis. In V1, the regions of interest (ROIs) were individually defined for each participant based on the data from the position experiments and a V1 mask that was individually obtained from the retinotopic mapping. This method was performed by contrasting all stimuli at one position with all other positions using a contrast threshold of p < 0.05 corrected with FDR and with a spatial extent of at least 10 voxels (Fig 3A in [20]). The neural activation to face and house images showed consisted position on the V1 cortex. In V1 area of each hemisphere, we defined 2 functional ROIs according neural activities of 2 positions of stimuli (face and house at positions of eccentricity 16° and 32°). The neural activities in response to the images of the faces or houses at each stimulus position were assigned as the BOLD response amplitudes in a matched ROI.

To avoid the double dipping, face-selective areas (FFA and OFA) and house-selective areas (PPA and TOS) of each participant were defined based on the mean activations from the 5 locations on the vertical meridian, including positions of eccentricity 0°, 16° and 32°. The FFA ROIs were defined as the regions that responded more strongly to images of faces than houses, and the house-selective areas (PPA and TOS) were identified as the regions that responded more strongly to images of houses than faces. The contrast threshold was p < 0.05, and the data were corrected for FDR with a spatial extent of at least 10 voxels. We extracted the response magnitude of each voxel in each ROI to the various conditions of the position scans and then used the mean response magnitude within a given ROI. The neural response magnitudes were similar across the left and right ROIs and were pooled across both hemispheres. The results from one hemisphere were treated as one sample. The signal changes were subjected to an analysis of variance (ANOVA) with repeated measures using SPSS software (version 16.0; SPSS Inc., Chicago, Ill).

### Multi-Voxel Pattern Analysis (MVPA)

Pattern classification analysis was executed using the raw intensity values. After the general preprocessing stage (including smoothing), the pattern classification data were detrended and normalized (z-score). This procedure was applied for the full-scan voxel time-courses. For each of the conditions, the mean intensity for the condition was subtracted from the intensity value of each voxel [28, 29]. This procedure was performed separately for the data from each scan to prevent information leakage in the cross-validation procedure. Additionally, the time-courses were shifted by two volumes (4 s) to account for hemodynamic lag. Thus, each scan consisted of one block per position condition with four data points (TRs). Across the six scans, the total number of data points per position and per category was 4 × 6 = 24. The leave-one-run-out cross-validation procedure was repeated 6 times, and the results were subsequently averaged. The contralateral and ipsilateral neural responses in each ROI underwent binary classifications for each position and category. We noted that the ROIs had both ipsilateral and contralateral responses to the image at 0°. Thus, there are no results related to the position at 0°.

The primary classification package was the LibSVM MATLAB implementation of the linear support vector machine (http://www.csie.ntu.edu.tw/~cjlin/libsvm/). The pattern classification analysis was performed using a custom MATLAB code. The classification results of each ROI were established above the chance level (0.5) by one-sample t-test (p < 0.05). The classification accuracies were similar across the left and right ROIs and were therefore pooled across both hemispheres. The results from one hemisphere were treated as one sample. Additionally, we found that the number of voxels in each ROI ranged from 10 to 230 with a mean of 80. We thus performed the MVPA using ten random voxels from all voxels of one ROI and repeated this process 100 times. The 100 time of classification accuracies for each ROI were averaged. The final classification accuracies were subsequently analyzed via repeated measures ANOVA.

## Results

### Behavior performances in a wide field

In the position experiments, behavior performances at each position are listed in Table 1 in S1 File. Some subjects had no or less response to the images of faces or houses at the most peripheral positions, and then resulted in significant lower than the chance levels (50%). The lower accuracy might indicate that the subject failed to discriminate the stimuli, and then we further analyzed the condition of eccentricities 0°to 32°. We found significant effect of eccentricity for the accuracy at right meridian positions (p < 0.05). The detailed statistical values are listed in Table 2 in S1 File. When the most peripheral positions were excluded, we only found significant effect of eccentricity for the accuracy at left meridian position. A pairwise comparison showed that the 32° positions had lower accuracy than the 16° positions (p < 0.05).

### Neural activity in response to contralateral and ipsilateral objects

**Fig 2:**
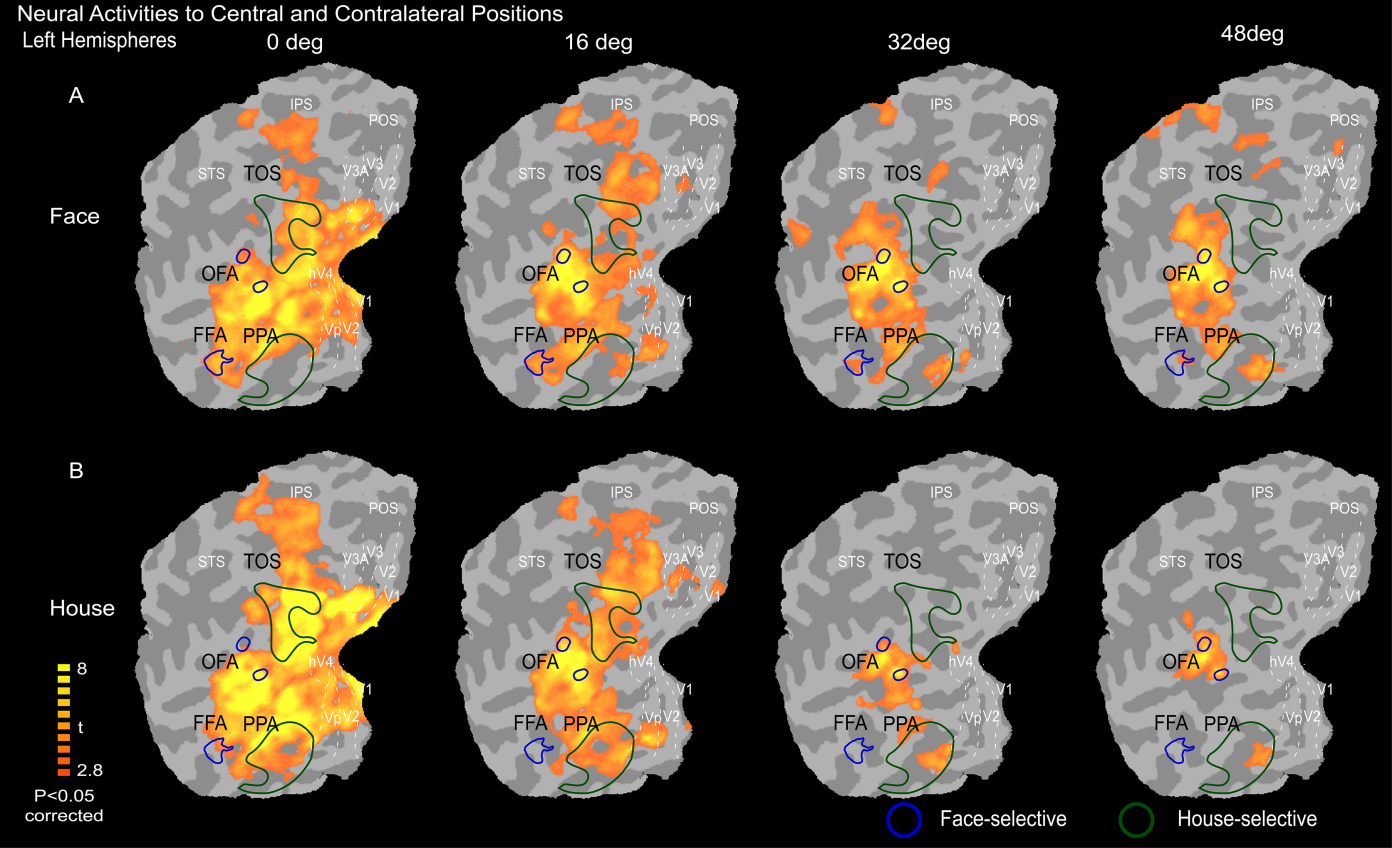
Mean neural activity maps to contralateral stimuli on a flat visual cortex. Here, the image in (A) shows the neural activity in response to contralateral face images, and (B) shows the neural activity in response to contralateral house images. The face-selective areas (FFA and OPA) and house-selective areas (PPA and TOS) were defined based on the mean activities of faces vs. houses (and houses vs. faces) from condition on the vertical meridian. The locations of V1, V2, V3, V3A, and hV4 are outlined by white dashed lines.

**Fig 3:**
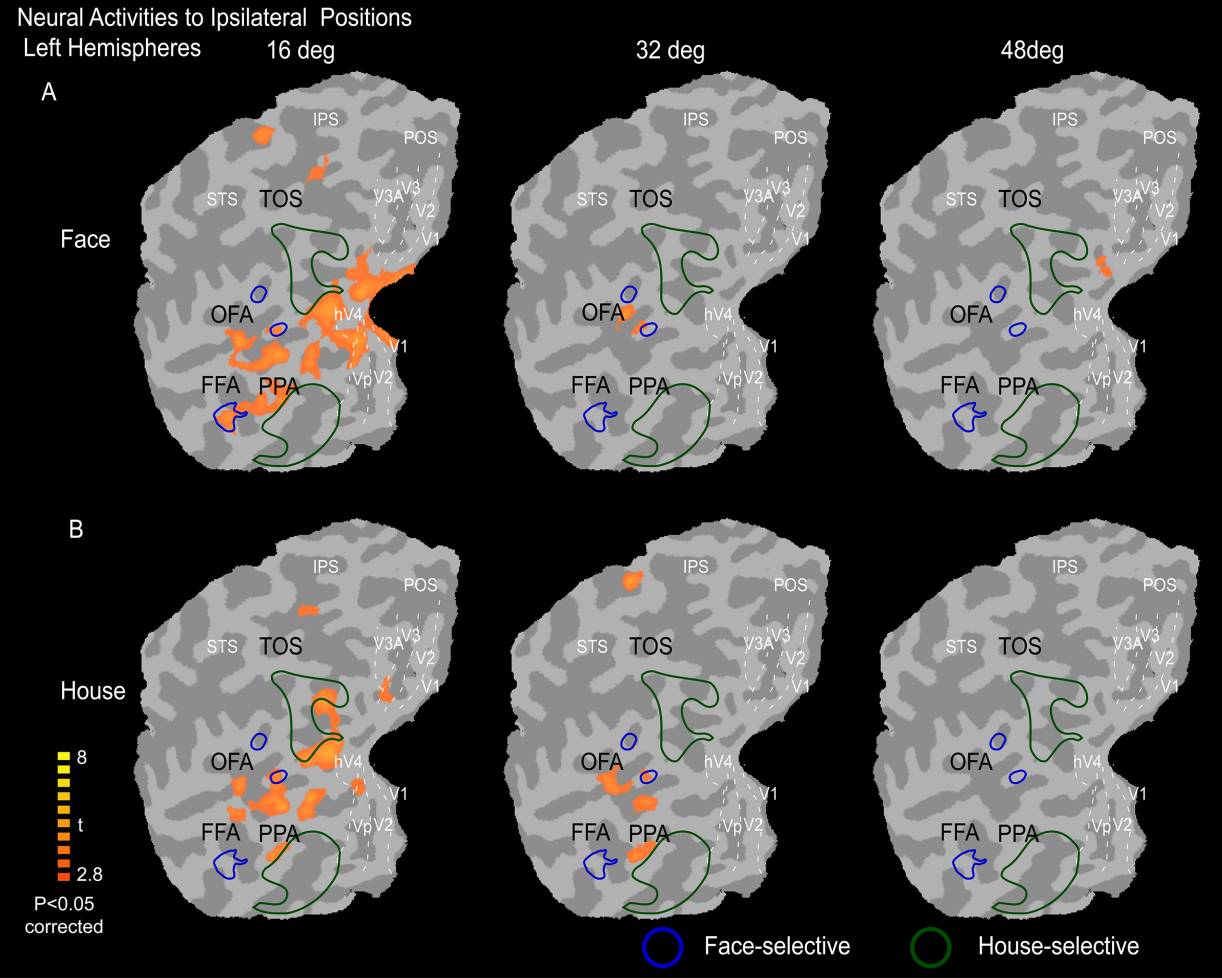
Mean neural activity maps to ipsilateral stimuli on a flat visual cortex. Here, the image in (A) shows the neural activity in response to ipsilateral face images, and (B) shows the neural activity in response to ipsilateral house images. The face-selective areas (FFA and OPA) and house-selective areas (PPA and TOS) were defined based on the mean activities of faces vs. houses (and houses vs. faces) from condition on the vertical meridian. The locations of V1, V2, V3, V3A, and hV4 are outlined by white dashed lines.

We present the activity maps on a flat visual cortex. The locations of V1, V2, V3, V3A, and hV4 are outlined by white dashed lines (Figs 2 and 3). The mean activity maps show the neural responses to face and house images presented in the contralateral visual field (Fig 2). The neural activity in the visual cortex was different with respect to eccentricity and category. Central positions elicited stronger neural activities than peripheral positions. Additionally, we also found that face-selective areas (FFA and OFA) had stronger neural responses to face stimuli than to house stimuli, while the reverse was true for house-selective areas (PPA and TOS). Generally, these results were consistent with the retinotopic organization of the visual cortex and category-selective activities that have been reported previously [20, 25, 26, 30, 31].

Fig 3 illustrates the mean activity maps show the neural responses to face and house images presented in the ipsilateral visual field. The early visual areas (V1-V3) exhibited no neural activity to ipsilateral face and house images. However, a weaker neural activity in response to ipsilaterally presented faces and houses was apparent within the higher-order visual areas, including large parts of the occipital and parietal cortices between V3A and hV4, and this activity was much weaker than the responses to contralateral stimuli along the eccentric positions. Most interestingly, we observed that the face-selective areas exhibited stronger neural activity in response to ipsilateral stimuli, whereas the house-selective areas exhibited weak or no neural activity to ipsilateral stimuli. Additionally, it is important to note that the intensity of the neural response decreased as the stimulus appeared further from the central fixation point, which is consistent with the neural response to contralateral stimuli.

### Neural response in V1

**Fig 4:**
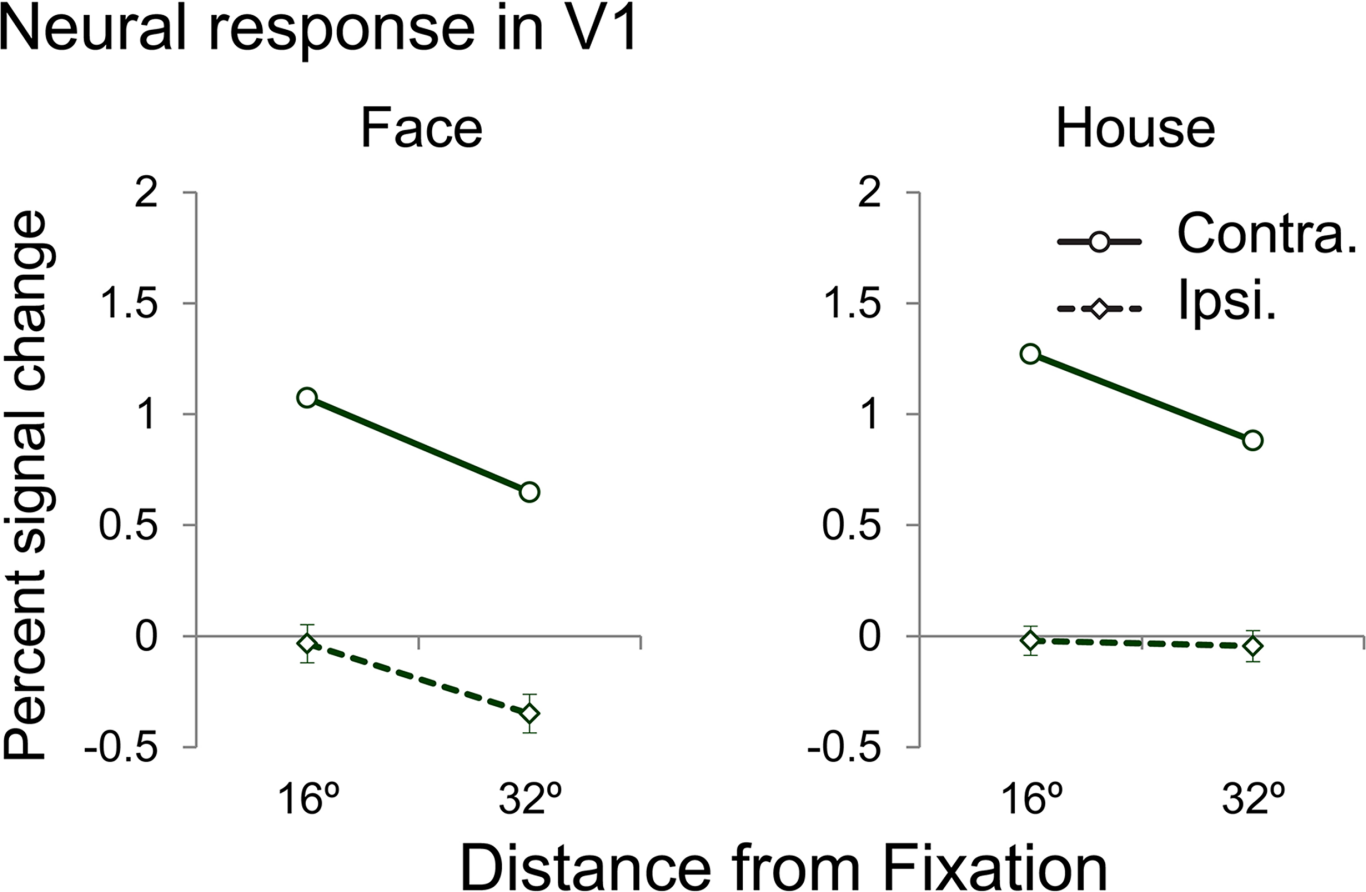
Neural response amplitudes to contralaterally and ipsilaterally presented stimuli in V1. (A) The neural responses to contralaterally and ipsilaterally presented faces, and (B) the neural responses to contralaterally and ipsilaterally presented houses.

The neural response magnitudes were pooled across both hemispheres. Fig 4 shows the neural responses to face and house images located at the contralateral and the ipsilateral positions. In V1, there were significant positive responses to contralateral face and house images at two eccentricity positions (16°and 32°; all p < 0.001, t-test) but negative and zero neural responses (i.e., no detectable response to ipsilateral stimuli with our stimulation protocol) to ipsilateral face and house images presented at the same two eccentricity positions. Additionally, the neural response amplitudes were significantly higher at the close eccentricity positions than the peripheral eccentricity positions (all: p < 0.01) for the contralateral face and house images but not for the ipsilateral face and house images (p > 0.4).

### Contralateral and ipsilateral neural responses to objects

**Fig 5:**
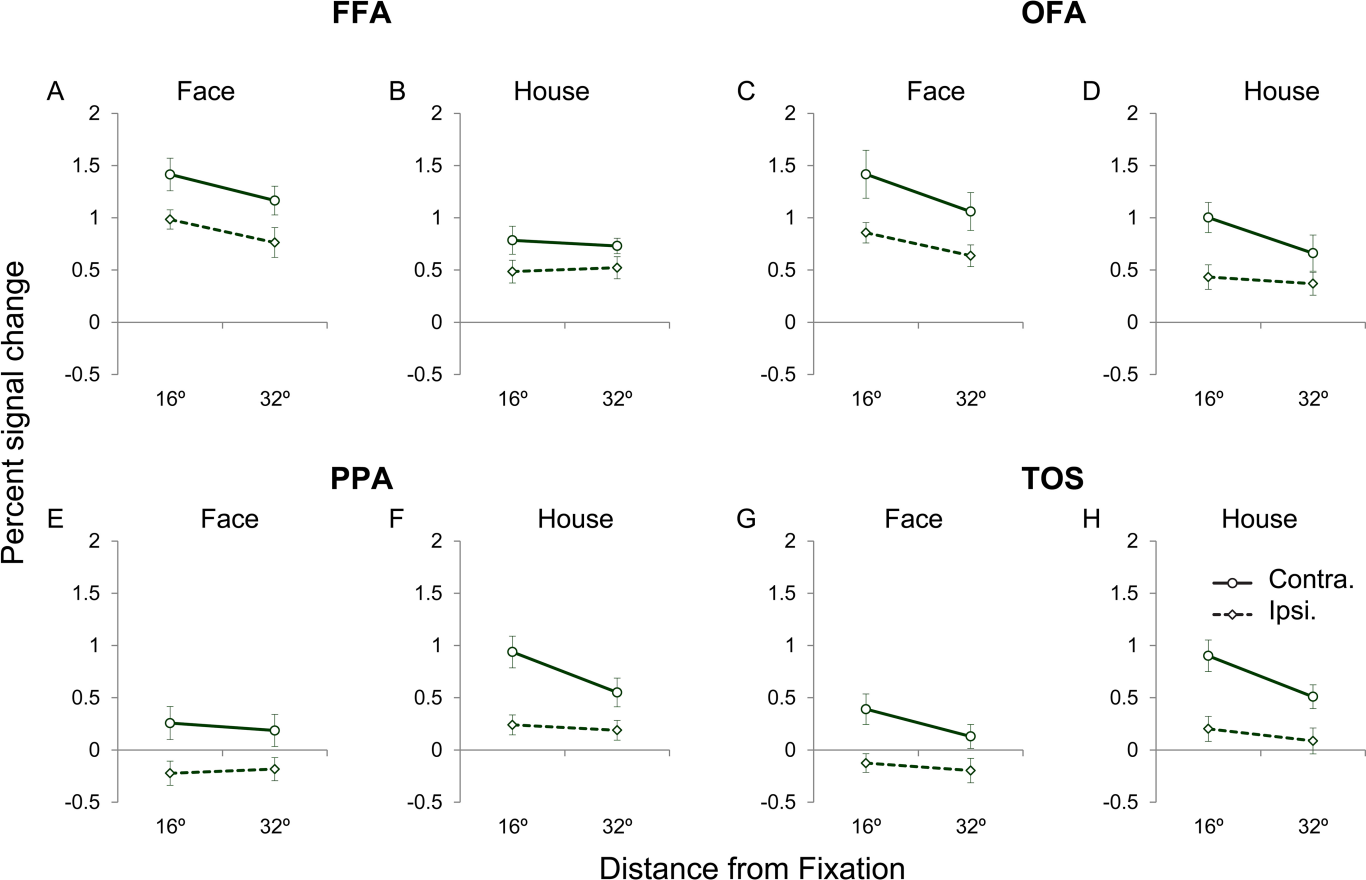
The neural response amplitudes to contralateral and ipsilateral stimuli in the face- and house-selective areas. These figures show the contralateral and ipsilateral responses in FFA (A and B), OFA (C and D), PPA (E and F) and TOS (G and H) to faces (A, C, E and G) and houses (B, D, F and H).

The neural response magnitudes are presented in Fig 5 and Table 3 in S1 File. When the face images (Fig 5A) and house images (Fig 5B) were presented in the contralateral and ipsilateral visual fields across the two eccentricity positions, there were significantly positive neural responses in the FFA and OFA (all: p < 0.01, t-test with Bonferroni correction for multiple comparison). In the house-selective areas (PPA and TOS), significantly positive neural responses were observed when the house images were presented contralaterally at the two eccentricity positions (all: p < 0.01, t-test with Bonferroni correction, Fig 5C). In contrast, ipsilaterally presented house images elicited no significant positive responses (all: p > 0.05, t-test with Bonferroni correction, Fig 5D). Neither the contralaterally or ipsilaterally presented face images, which were not the preferred stimulus category for the PPA and TOS, elicited no significant positive neural responses (all: p > 0.05, t-test with Bonferroni correction, Fig 5D).

In the four investigated areas, three-way repeated measures ANOVAs with the effects of category, visual field and eccentricity were applied to analyze the neural responses and revealed significant main effects of category [all: F(1, 13) > 21.67, p < 0.001]. There were significant main effects of eccentricity in OFA and TOS [all: F(1, 13) > 10.66, p < 0.006]. The Table 4 in S1 File shows the results of the ANOVAs. Post hoc pairwise t-tests with Bonferroni correction for multiple comparisons were performed. We found that the FFA and OFA exhibited stronger activation to face images than to house images (p < 0.05) in the contralateral visual field, whereas the ipsilateral neural responses to faces were larger than only those to houses at the 16° and 32° eccentricities (p < 0.05). In the PPA and TOS, the house images elicited stronger activation than the face images (p < 0.05) in the contralateral and ipsilateral visual fields at the eccentricities of 16° and 32°.

Additionally, we also found significant main effects of the visual field in four areas [all: F(1, 13) >12.76, p < 0.003] (Fig 5, Table 4 in S1 File). Post h*oc* pairwise t-tests with Bonferroni correction for multiple comparisons were performed. Generally, the results revealed larger differences between the contralateral and ipsilateral neural responses. The neural responses to the contralateral face images were significantly larger than the ipsilateral neural responses at the 16° and 32° eccentricities (p < 0.05) in the FFA and at the 16° eccentricity (p < 0.05) in the OFA. Larger differences between the contralateral and ipsilateral neural responses were found in the house-selective areas. The neural responses to contralateral house images were significantly larger than the ipsilateral neural responses at two eccentricities (p < 0.05) in the PPA and TOS. For the non-preferred objects, there were significant differences between the contralateral and ipsilateral neural responses at the 16° eccentricity in the OFA, PPA and TOS (p < 0.05).

### Classification performance

**Fig 6:**
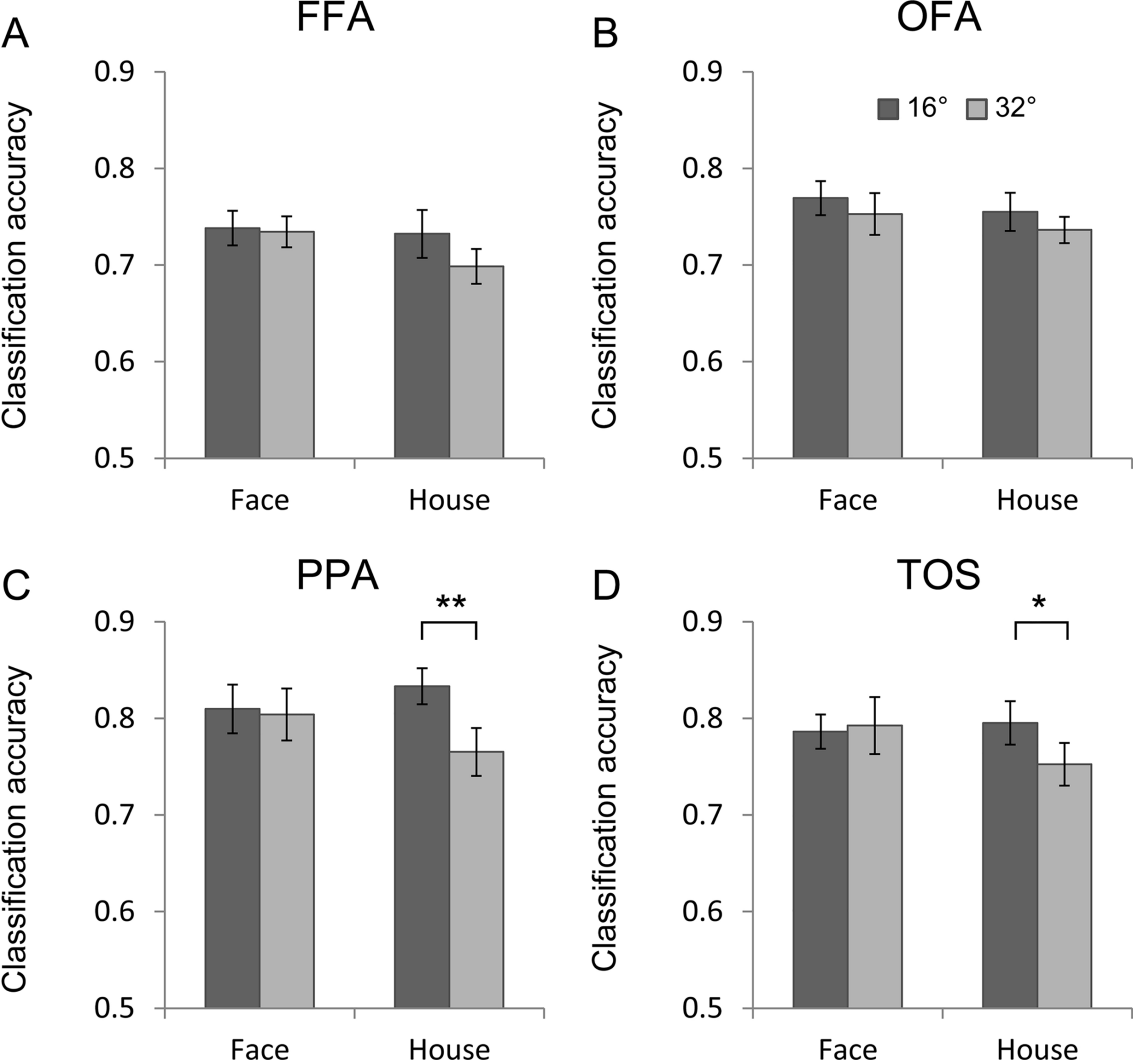
The classification performances of the neural responses to stimuli in the contralateral and ipsilateral visual fields. (A, B) The classification performances in the face-selective areas (FFA and OFA). (C, D) The classification performances in the house-selective areas (PPA and TOS). *: p < 0.05, **: p < 0.01, Bonferroni corrected.

To further identify differences between the neural activities in the contralateral and ipsilateral visual fields, we examined the classification performance of the MVPA between the contralateral and ipsilateral neural responses. A higher accuracy of the classification performance indicated a greater difference between the contralateral and ipsilateral visual fields. The ipsilateral and contralateral responses could not be separated at the eccentricity of 0°; thus, we reported the classification performance from 16° and 32°. The voxel numbers and classification accuracies were no differences between left and right ROIs (p >0.1), and the classification accuracies were therefore pooled across ROIs in both hemispheres. The mean classification accuracies are presented in Fig 6. In the face- and house-selective areas, the classification accuracies at all eccentricities were significantly larger than chance (p < 0.01, permutation test with 1000 permutations, corrected by Bonferroni), which indicated a significant difference between the ipsilateral and contralateral responses.

We first performed a three-way ANOVA with area (FFA, OFA, PPA, and TOS), stimulus category (face and house) and eccentricity as the repeated measures. The results revealed significant main effects of area [F (3, 39) = 7.93, p = 0.01] and eccentricity [F (1, 13) = 5.69, p = 0.03]. Post hoc pairwise comparisons revealed that the classification accuracies in the FFA were smaller than those in the PPA and TOS (t-tests, p < 0.05, Bonferroni corrected Fig 6). The PPA and TOS exhibited larger classification accuracies at the eccentricity of 16° than the eccentricities of 32°for the house images (t-test, p < 0.05, Bonferroni corrected).

## Discussion

In the present study, we investigated the neural responses to contralateral and ipsilateral images in face- and house-selective areas. For the lower behavior performances and rather weak neural activities at most peripheral positions, we main focused on the positons of eccentricity of 16° and 32° in the following ROI analysis. The face-selective areas exhibited intense neural responses to ipsilaterally presented face images and non-preferred images (house), whereas the PPA and TOS exhibited substantially smaller neural responses to the ipsilaterally presented images and negative or zero responses to the face images (non-preferred object). We also found that the face- and house-selective areas exhibited neural responses to the ipsilateral images that were smaller than the responses to the contralateral images, particularly at eccentricities of 16° and 32°. Moreover, the classification accuracies for the contralateral and ipsilateral neural responses were also determined. The classification accuracies in FFA were smaller than those in PPA and TOS. The classification accuracies exhibited a significant effect of eccentricity in the PPA and TOS.

### The difference in ipsilateral responses in object-selective areas

The lateral and ventral visual cortices exhibited obvious neural responses to checker boards [4] and moving dots [32, 33], as well as to faces [14], objects [14, 16] and senses [15] in the ipsilateral field. These ipsilateral responses were always weaker than the corresponding contralateral responses [13–16, 34]. The magnitude of neural responses to ipsilateral stimuli differed across the visual cortex [13–15, 34]. In the present study, we employed stimuli in a wide-view field and found obvious ipsilateral responses in the face- and house-selective areas (Fig 5), which is consistent with previous findings.

Interestingly, we revealed that the face-selective areas exhibited significant responses to ipsilaterally presented face and house images even when presented in the far peripheral position (Fig 5A). The ipsilateral responses tended to decrease as the eccentricity of the stimulus increased, which was similar to the contralateral responses. However, the house-selective areas exhibited only slight responses to houses presented ipsilaterally at 16° of eccentricity and no or smaller ipsilateral responses to the same stimuli when presented in a more wide-view field (Fig 5B). At the central position, there were stronger neural responses to the ipsilaterally presented stimuli in the FFA than in the PPA, which is consistent with the findings of previous reports that presented stimuli only in the central areas of the visual field [13, 14]. These findings implied that these face- and house-selective areas exhibited different ipsilateral responses.

Furthermore, we used classification accuracy to evaluate the differences between the neural responses to contralateral and ipsilateral stimuli. A higher accuracy of the classification performance indicated a greater difference between the contralateral and ipsilateral visual fields. The classification performances were significantly above the chance level, which is consistent with the findings of previous studies [35, 36]. We found that the classification accuracies in the FFA were smaller than those in the PPA and TOS. This finding implied smaller differences between contralateral and ipsilateral neural responses, which were apparent from the percent signal change result. Previous studies have also reported that the FFA exhibits larger ipsilateral neural responses than other category-selective areas [14, 37]. These findings suggest that the FFA regions receive different retinotopic inputs and have a different visual processing hierarchy compared with the PPA and TOS.

As the eccentricity increased, the differences between the contralateral the ipsilateral responses became smaller. The different trends toward a decrease in the response as the eccentricity of the presented stimuli increased might be related to the Cortical magnification of the visual cortex, which decrease as the eccentricity increasing [18, 20]. Importantly, the classification accuracies also exhibited a decrease as the eccentricity increased in the house-selective areas (PPA and TOS), which means that the differences between the contralateral and ipsilateral response became smaller. We found that the contralateral responses exhibited a sharp decreasing trend, and the ipsilateral responses exhibited a smaller decreasing trend as the eccentricity increased. Thus, we proposed that the contralateral and ipsilateral PPA and TOS responses exhibited different topological structures in the processing of the central and peripheral visual fields. In contrast, for the FFA and OFA, the contralateral and ipsilateral responses had similar topological structures for the processing of the central and peripheral visual fields.

### A potential neural mechanism for ipsilateral responses

These ipsilateral responses could be explained either by neurons with large receptive fields that are centered in the contralateral field but extend across the vertical meridian or by neurons with small receptive fields that tile across both visual fields [15, 38–40]. The face-selective areas (FFA and OFA) exhibited significant responses to the wide-field ipsilateral presentation of face and house images. The distance between the stimulus locations and the center of fixation were 16° and 32°, which are larger than the size of the population receptive fields (8° to 10° at an eccentricity of 10°)of voxels on the lateral and ventral occipital cortex and fusiform gyrus [21–24]. Our finding implies that the receptive fields do not extend across the vertical meridian because the population receptive fields in the face-selective area did not cover the ipsilateral visual field at large eccentricities. Instead, the ipsilateral responses could be attributed to the activation of neurons with small receptive fields that tiled over the ipsilateral visual field [17]. Indeed, single-unit recording studies have provided evidence that the receptive fields of neurons can differ in size and that these neurons have “hot spots” [41]. Neuroimaging studies have also showed that the receptive fields in the higher level areas, such as LO, VO, and MT+, extend significantly into the ipsilateral visual field [39, 40]. However, in V1–V3 the receptive fields are confined largely to the contralateral visual field [39]. The ipsilateral receptive fields in higher level areas could be considered as the bottom-up connections from lower level areas to object-selective higher level areas. Shigihara et.al (2014) used magnetoencephalography (MEG) to analyze the time course of the earliest responses to face and house stimuli, and found that both face and house stimuli presented in the left and right hemispheres field activated both striate cortex and the prestriate cortex with a peak at around 40 ms after stimulus onset, suggested a parallel strategy in addition to the hierarchical strategy for form perception[42]. Moreover, the classification accuracies in these category-selective areas also support the explanation that the receptive fields are over the ipsilateral visual field. It is more likely that the ipsilateral responses in both the face- and house-selective areas relied on a similar mechanism. By presenting the stimuli using a wide-view field, our findings suggest that neurons in the face- and house-selective areas have small receptive fields that are tiled over the ipsilateral visual field to arouse slight ipsilateral neural activities.

The corpus callosum is widely perceived as the most plausible anatomical candidate for mediating interhemispheric transmission [43, 44]. This functional connectivity is likely to be mediated by the corpus callosum because damage to this commissure dramatically reduces correlated magnetic resonance activity across the hemispheres [45]. Psychophysiological interaction analysis has indicated a link between the activation of the right FFA and the right face-selective areas (the right FFA and OFA) [37]. Davies-Thompson et al. also found evidence for significant functional connectivity between the core face-selective regions, particularly between the OFA and FFA, and significant interhemispheric correlations between corresponding face regions [46]. However, there is currently no evidence for interhemispheric connectivity between the face areas. Using DTI in connection with fiber tractography, several studies have identified occipital–callosal fiber tracts that pass through the splenium of the corpus callosum and connect occipital labels in the two hemispheres [47–50]. Gschwind et al. found connections between the early visual areas and OFA, and the early visual areas, OFA, and FFA participate in the same cortico-cortical network that is associated with the inferior longitudinal fasciculus [51, 52]. Our present results and previous studies [4, 5] have demonstrated the abrupt increase of ipsilateral activity in the areas anterior to areas V3a and V4v, which may be the locus of convergence. Thus, from area V1 up to the level of areas V3a and V4v, the information from the two visual hemifields may separately go through different encoding processes in different hemispheres and converge at least after the lateral occipital region. In the present study, the neural responses to ipsilateral stimuli in the face-selective areas were significantly positive and comparable to the neural responses to the stimuli in the contralateral visual field. These face-selective areas in the two hemispheres formed a functional network [37, 46] via interhemispheric communication through intrahemispheric connectivity of white matter connection [51, 52] and interhemispheric connectivity via the corpus callosum and occipital white matter connection [47–50].

In the house-selective areas, a significant ipsilateral response was observed only at an eccentricity position of 16° and was absent at a more peripheral position. The house-selective areas are likely to have weaker interhemispheric communication than the face-selective areas, particularly for the far peripheral visual field. According to the interhemispheric communication via the corpus callosum [47–50], the house-selective areas might have fewer connections with the regions with interhemispheric connections.

### The preference of category

Previous studies [11, 12] have found that the neural responses in category-selective areas exhibit a preference of category. Responses to the preferred category were significantly greater than those to the second-highest category. In the present study, we also found that the neural responses to the contralateral stimuli exhibited preferences of category in the FFA, OFA, PPA and TOS, and these findings are corroborated by previous studies [6–12]. We also found that those preferences of category became slightly weaker when the stimuli were located in the peripheral visual field. Although we adopted a very easy object recognition task to make sure the subjects could attend to the whole visual field and fixate the fixation point, the results revealed lower recognition performances in the peripheral eccentricities (Table 1 in S1 File). The neural activity decoding could fail to classify objects if the subject does not perceive them. Thus, we main focused on the results from the eccentricity 16° and 32°, to make the founding more reliable. With regard to the ipsilateral stimuli, we also found a weaker preference of category in the peripheral visual field. Additionally, we should note that the neural responses to faces and houses in ipsilateral visual field were significantly weaker than the neural responses of contralateral visual field, especially in the house-selective areas. These findings suggested that, in general, the object-selective areas might have similar topological structures for the processing of ipsilateral visual information, but the neural information flow from the intrahemispheric visual cortex was much smaller than that of the interhemispheric visual cortex.

## Conclusion

We investigated the neural responses elicited in face- and house-selective areas by ipsilaterally presenting stimuli. Interestingly, the face-selective areas exhibited intense neural responses to the ipsilaterally presented face images and non-preferred images (houses), whereas the PPA and TOS exhibited substantially smaller neural responses to the ipsilaterally presented images. Compared with the contralateral neural responses, the preference of category of the ipsilateral neural responses was smaller. We also found that the face- and house-selective areas exhibited neural responses to the ipsilateral images that were smaller than the responses to the contralateral images, particularly at the eccentricity of 16°. In the PPA and TOS, the closer eccentricity elicited greater classification accuracies than those in peripheral eccentricity. We propose that these ipsilateral neural responses might be interpreted by interhemispheric communication through intrahemispheric connectivity of white matter connection and interhemispheric connectivity via the corpus callosum and occipital white matter connection. Furthermore, the PPA and TOS likely have weaker interhemispheric communication than the FFA and OFA, particularly in the peripheral visual field.

## Supporting information

S1 File The supporting information of result. (DOCX)

S2 File The neural response amplitudes to contralateral and ipsilateral stimuli in face- and house-selective areas. (XLSX)

S3 File The classification performances of the neural responses to stimuli in the contralateral and ipsilateral visual fields. (XLSX)
