# Supplementary material for: Differences in neural responses to ipsilateral stimuli in wide-view fields between face- and house-selective areas
Source: PLoS One. 2018 Feb 16;13(2):e0192532. doi: 10.1371/journal.pone.0192532 (PMC5815592; doi:10.1371/journal.pone.0192532)
Supplement: S1 File — (DOCX) [file pone.0192532.s001.docx]

**The supporting information of result**

Table 1. Mean behavioral performance during object experiments.

| Position | | Response time (ms) | | | | Percentage accuracy | | | |
| --- | --- | --- | --- | --- | --- | --- | --- | --- | --- |
| Meridian | Eccentricity | Faces | | Houses | | Faces | | Houses | |
| Central | 0º | 632 | ±26 | 611 | ±44 | 83 | ±3 | 83 | ±3 |
| Left horizontal | 16º | 628 | ±52 | 636 | ±47 | 90 | ±3 | 91 | ±4 |
|  | 32º | 640 | ±55 | 568 | ±52 | 80 | ±4 | 75 | ±4 |
|  | 48º | 687 | ±66 | 692 | ±66 | 28 | ±6 | 14 | ±6 |
| Right horizontal | 16º | 544 | ±57 | 528 | ±59 | 77 | ±3 | 80 | ±2 |
|  | 32º | 566 | ±43 | 546 | ±54 | 83 | ±6 | 91 | ±6 |
|  | 48º | 503 | ±30 | 575 | ±37 | 71 | ±7 | 70 | ±7 |
| Upper vertical | 16º | 557 | ±54 | 581 | ±59 | 88 | ±3 | 91 | ±2 |
|  | 32º | 602 | ±72 | 656 | ±67 | 66 | ±5 | 69 | ±3 |
|  | 48º | 774 | ±63 | 682 | ±63 | 8 | ±5 | 17 | ±5 |
| Lower vertical | 16º | 559 | ±39 | 619 | ±38 | 88 | ±6 | 89 | ±5 |
|  | 32º | 628 | ±23 | 605 | ±21 | 77 | ±6 | 65 | ±7 |
|  | 48º | 695 | ±48 | 746 | ±48 | 21 | ±8 | 24 | ±8 |

Values are represented as the means ± SEM.

Table 2. The ANOVA result of behavior performance of eccentricity 0º - 32º

|  | | Response time (ms) | | Percentage accuracy | |
| --- | --- | --- | --- | --- | --- |
|  | | F | Sig | F | Sig |
| Right horizontal meridian | Eccentricity | 1.68 | 0.23 | 1.18 | 0.34 |
|  | Category | 0.37 | 0.57 | 1.85 | 0.22 |
|  | Eccentricity*Category | 0.002 | 0.99 | 1.04 | 0.38 |
| Left horizontal meridian | Eccentricity | 0.26 | 0.77 | 5.31 | **0.02** |
|  | Category | 0.47 | 0.52 | 0.28 | 0.61 |
|  | Eccentricity*Category | 0.59 | 0.57 | 0.54 | 0.6 |
|  | Eccentricity | 0.71 | 0.51 | **8.06** | **0.006** |
| Upper vertical meridian | Category | 0.49 | 0.51 | 0.53 | 0.49 |
|  | Eccentricity*Category | 0.43 | 0.66 | 0.51 | 0.95 |
|  | Eccentricity | 0.68 | 0.52 | **10.95** | **0.02** |
| Lower vertical meridian | Category | 0.04 | 0.86 | **8.28** | **0.03** |
|  | Eccentricity*Category | 0.47 | 0.64 | **4.74** | **0.03** |

The significant effects were indicated by bold letter.

Table 3. The mean neural response amplitudes to contralateral and ipsilateral stimuli in face- and house-selective areas.

| Areas | Visual field | preferred objects | |  | non-preferred objects | |
| --- | --- | --- | --- | --- | --- | --- |
|  |  | deg 16 | deg32 |  | deg 16 | deg32 |
| FFA | contralateral | 1.41(0.16) | 1.17(0.14) |  | 0.78(0.13) | 0.73(0.07) |
|  | ipsilateral | 0.98(0.09) | 0.76(0.14) |  | 0.49(0.11) | 0.52(0.10) |
| OFA | contralateral | 1.42(0.23) | 1.06(0.18) |  | 1.01(0.14) | 0.66(0.17) |
|  | ipsilateral | 0.86(0.10) | 0.64(0.10) |  | 0.43(0.12) | 0.37(0.11) |
| PPA | contralateral | 0.94(0.15) | 0.55(0.14) |  | 0.26(0.16) | 0.19(0.15) |
|  | ipsilateral | 0.24(0.10) | 0.19(0.09) |  | -0.22(0.12) | -0.18(0.11) |
| TOS | contralateral | 0.90(0.15) | 0.51(0.11) |  | 0.39(0.15) | 0.13(0.11) |
|  | ipsilateral | 0.20(0.12) | 0.09(0.12) |  | -0.13(0.09) | -0.20(0.12) |

Values are represented as the means ( SEM).

Table 4. The ANOVA result of neural response amplitudes

|  | FFA | | OFA | | PPA |  | TOS |  |
| --- | --- | --- | --- | --- | --- | --- | --- | --- |
|  | F | p | F | p | F | p | F | p |
| Visual field | **25.41** | **< 0.001** | **12.76** | **0.003** | **13.57** | **0.003** | **20.68** | **0.001** |
| Category | **74.54** | **< 0.001** | **39.98** | **< 0.001** | **112.93** | **< 0.001** | **21.67** | **<0.001** |
| Eccentricity | 3.14 | 0.10 | **35.89** | **< 0.001** | 3.89 | 0.07 | **10.66** | **0.006** |
| Visual field * Category | 1.15 | 0.31 | 0.16 | 0.72 | 0.38 | 0.55 | 1.08 | 0.32 |
| Visual field * Eccentricity | 0.22 | 0.64 | **5.09** | **0.04** | 2.72 | 0.12 | 2.98 | 0.11 |
| Category * Eccentricity | 2.28 | 0.15 | 0.53 | 0.48 | 2.74 | 0.12 | 0.59 | 0.45 |

The significant effects were indicated by bold letter.
